# Dengue virus seroprevalence study in Bangphae district, Ratchaburi, Thailand: A cohort study in 2012-2015

**DOI:** 10.1371/journal.pntd.0010021

**Published:** 2022-01-04

**Authors:** Kriengsak Limkittikul, Pornthep Chanthavanich, Kang Sung Lee, Jung-Seok Lee, Supawat Chatchen, Sl-Ki Lim, Watcharee Arunsodsai, In-Kyu Yoon, Jacqueline Kyungah Lim

**Affiliations:** 1 Department of Tropical Pediatrics, Faculty of Tropical Medicine, Mahidol University, Bangkok, Thailand; 2 International Vaccine Institute, Seoul, Republic of Korea; 3 Coalition for Epidemic Preparedness Innovations, Washington, DC, United States of America; DoD - AFHSB, UNITED STATES

## Abstract

**Background:**

To determine the seroprevalence and transmission dynamics of dengue virus (DENV), age-stratified longitudinal serological surveys were conducted in Bangphae district, Ratchaburi province, Thailand, for 3 years between April 2012 and April 2015.

**Methodology:**

The surveys enrolled 2012 healthy children and adults between 1 and 55 years-of-age, and a longitudinal serosurvey of six repeated bleeds of the same cohort of individuals was conducted every 8 months for the first 2 years (M0, M8, M16) and every half a year (M24, M30, M36) for the rest of the study period. All samples were tested using in-house indirect sandwich dengue IgG ELISA to determine DENV antibody titer, and 640 paired samples which showed rising of DENV IgG titers in paired serum were further tested using in-house neutralization assay, Plaque Reduction Neutralization Test (PRNT_50_).

**Principal findings:**

When compared against the gold standard based on the results of PRNT_50_, sensitivity and specificity of indirect ELISA were found to be both about 85%. The overall DENV IgG positivity determined by ELISA was 74.3% in 2012 and increased to 79.4% by the final sample collection in 2015. In our study sample, more than 98% of subjects older than 25 years were found to be seropositive. Among 518 IgG negative subjects at enrollment, the seroconversion rates were measured in paired bleeds; the rates (between successive visits, approximately 6 months) ranged between 4.8% (between M16 and M24) and 14.7% (between M0 and M8). The dominant serotype of primary DENV infection cases based on seroconversion was identified from the PRNT results and it was DENV-2.

**Conclusions:**

Our study documented high levels of seroprevalence and rate of transmission. Given the importance of the serostatus and disease burden in consideration for dengue vaccine introduction, our data could be used in decision-making on implementation of various dengue control and preventive measures.

## Author summary

To estimate the proportion of individuals with past exposure to dengue virus (DENV), we conducted repeated serological surveys in Bangphae district, Ratchaburi province, Thailand, between April 2012 and April 2015. About 45% of the subjects were under 15 years-of-age. The surveys enrolled 2012 healthy individuals between 1 and 55 years-of-age, and a longitudinal serosurvey of six repeated bleeds of the same cohort of individuals was conducted approximately every 6 months. All samples were tested using an indirect dengue IgG ELISA and a subset of paired samples which showed rising of IgG values in pairs were tested using neutralization assay, used as the gold standard. When compared against the gold standard, sensitivity and specificity of indirect ELISA were found to be both 85%. The proportion of the study population with past DENV infection measured by IgG ELISA was 74.3% in 2012 and increased to 79.4% by 2015. More than 98% of subjects older than 25 years showed to have past exposure to dengue. Among 518 subjects without past exposure, the rate of getting infection was between 4.8–14.7% (between successive visits, approximately 6 months). The dominant serotype was DENV-2. The level of proportion of individuals with past DENV infection is an important consideration, and our data could be used in decision-making for dengue vaccine introduction.

## Introduction

Dengue, a mosquito-borne flavivirus infection caused by 4 antigenically distinct dengue viruses, is a major cause of mortality and morbidity in tropical and subtropical regions [1,2]. With a dramatic increase in recent decades, there are approximately 390 million infections reported each year worldwide, with 500,000 cases of severe dengue requiring hospitalization and 20,000 deaths [3–6].

Similar to other dengue-endemic countries, Thailand has been experiencing increased burden of dengue, despite mosquito control efforts, in both incidence and range of geographical distribution throughout the country [7,8]. Dengue Hemorrhagic Fever (DHF) was known to be typically confined to children in Asia, however its trend is changing with increased modal age and more cases among young adults [9–11]. In Thailand, with a well-established national dengue surveillance system, the provincial incidence rates are documented to be up to 698/100,000 person-years, reported in 2008 [12].

Given the documented burden of dengue in Thailand as well as other countries at risk, there is a need for effective and safe vaccines against dengue. Vaccine development is in progress, and the first dengue vaccine from Sanofi Pasteur, Dengvaxia, was licensed in 20 countries in Latin America and Asia, including Thailand [13]. However, this vaccine has a restricted indication in previously DENV-infected individuals from 9 years and older, due to increased risk of severe dengue in seronegative individuals [14,15]. Furthermore, WHO issued their position with one of the recommendations to perform pre-vaccination screening to identify persons with evidence of a past DENV infection [14,15].

In addition to a well-established national dengue surveillance system, much evidence is available on dengue from various field studies in Thailand [16–19]. However, the currently available vaccine is recommended only in individuals with prior DENV infection and, therefore, there is a growing need to determine serological prevalence of DENV and assess performance of diagnostic assay to obtain accurate serostatus information, which has become more relevant for consideration of pre-vaccination screening approach. Dengue Vaccine Initiative (DVI), in collaboration with Mahidol University, had conducted a longitudinal serosurvey of six repeated bleeds following the same individuals in Bangphae district, Ratchaburi province, Thailand during 2012–2015. The study targeted 2 objectives. First, we measured the population-level seroprevalence in the study area. Second, although it is an in-house assay and may be limited for future use in a commercial setting, we compared the performance of an indirect IgG ELISA against the gold standard, plaque reduction neutralization test (PRNT), to document its diagnostic accuracy in determining DENV serostatus.

## Methods

### Ethics statement

The study protocol received ethical approvals from the Institutional Review Boards (IRBs) of IVI (2011–007), the Ethics Committee of the Faculty of Tropical Medicine, Mahidol University (MUTM2011-031-06), and the Ethical Review Committee in Human Subjects (Ref.no.31/2554) of Thai MoPH. Written consent forms were obtained from each participant. If the subject is between 8 to 18 years old, a written assent form from the subject and a written informed consent from at least one parent or guardian were obtained. If subject is <8 years old, a written informed consent was obtained from at least one parent or guardian. If the subject is >18 years old, a written informed consent was obtained from the subject.

A longitudinal serosurvey of six repeated bleeds—every 8 months for the first 2 years (M0, M8, M16) and every half a year for the rest of the study period (M24, M30, M36)—was conducted for a 3-year study-period during 2012–2015 based on the same cohort of individuals. The age-stratified sample of approximately 2000 residents between 1 and 55 years-of-age reflected age distribution of the general population of Bangphae district, Ratchaburi province, Thailand, with about 45% of the enrollees under 15 years-of-age.

### Sample size calculation

In the target study area with the estimated population of 50,000 residents in Bangphae, the sample size for the serosurvey cohort was calculated based on the ability to detect the annual sero-incidence of DENV, reported to range between 5% (adults 35 years and older) and 17% (children under 15 years) from a previous study [20], with 95% confidence level and 5% absolute precision. Then, the estimated sample size was adjusted by 30% to allow for possible drop-out rate, resulting in an estimated sample size of approximately 2,000 individuals.

### Subject recruitment and study procedure

Clusters were defined as villages of about 750 residents. In Bangphae district, there are 65 villages, with 15 of them with more than 1000 residents. The villages with more than 1000 residents were split into 2 clusters to meet the cluster size of about 750 village residents. With a total of 80 clusters, 40 were randomly chosen, including semi-rural villages as well as more urban villages with promotion hospitals (health stations with primary care capabilities serving villages) and schools. Based on existing census information (village maps), households were randomly selected in the pre-selected 40 clusters. Household members (more than one member) of pre-selected households were invited for study participation, with approximately 50 individuals enrolled per cluster.

The enrollment criteria included individuals between 1–55 years of age who agreed to participate in the study, without major organ diseases or medication/disease which induced immunocompromised condition. After the enrollment, a blood draw of 5 ml was performed by study staff, and a short interview was conducted at every visit to complete data collection forms on episodes of fever and history of signs and symptoms during the interval, to check for possible association with silent DENV infection or mild dengue. All of the samples collected over six visits were tested using indirect sandwich dengue IgG ELISA and DENV serostatus was evaluated based on the IgG ELISA results.

Follow-up visits were arranged after subjects’ receipt of results. Without new recruitment, the same procedures were followed with the same subjects for the subsequent serosurveys. At subsequent visits, a question was asked in the serosurvey questionnaire whether the subject had a fever since the last serosurvey during the interval (Table 1). Also, there was a question asked in the serosurvey questionnaire whether the subject had a previous DENV infection (self-reported by the subject and not verified with information on medical charts). Consecutive blood samples which had rising ELISA antibody titers compared to the titers from preceding bleeds were subjected to 50% DENV plaque reduction neutralization test (PRNT_50_) for evaluation of appropriate cut-off point for seropositivity of indirect sandwich dengue IgG ELISA.

In the same target study area in Bangphae during an overlapping study period, we also conducted a fever surveillance study, with a separate consenting process, enrolling patients aged 1–55 years who presented with non-localized febrile illness at Bang Phae Community Hospital (BPCH) in Ratchaburi province, from October 2011 to September 2016 [21]. Paired blood samples were taken 10–21 days apart from enrolled patients and collected acute/convalescent sera were tested with in-house ELISA dengue IgM/IgG [21,22], and a subset (all of the serological positive cases with a small number of serological negative cases) with RT-PCR [21,23].

**Table 1:** Demographic characteristics of subjects by visit during the repeated (n = 6) serosurveys.

Time period	Visit 1	Visit 2	Visit 3	Visit 4	Visit 5	Visit 6
	M0 (Apr—May 2012)	M8 (Nov–Dec 2012)	M16 (Aug–Sep 2013)	M24 (Feb–Apr 2014)	M30 (Sep–Oct 2014)	M36 (Feb–Apr 2015)
**Number of subjects**	2012	1968	1933	1897	1842	1814
**Male: Female**	903:1109	880:1088	850:1083	826:1071	800:1042	790:1024
	(0.81:1)	(0.81:1)	(0.78:1)	(0.77:1)	(0.76:1)	(0.77:1)
**Sub-district of residence (n; %)**						
Wang-Yen	348 (17.3)	341 (17.3)	334 (17.3)	319 (16.8)	318 (17.3)	307 (16.9)
Bang-Phae	301 (15.0)	294 (15.0)	291 (15.1)	292 (15.4)	284 (15.4)	275 (15.2)
Wat-Kaeo	250 (12.4)	247 (12.6)	243 (12.6)	231 (12.2)	228 (12.4)	222 (12.2)
Hau-Pho	254 (12.6)	248 (12.6)	242 (12.5)	236 (12.4)	227 (12.4)	225 (12.4)
Don-kha	152 (7.6)	148 (7.5)	150 (7.8)	147 (7.7)	145 (7.9)	143 (7.9)
Don-Yai	203 (10.0)	201 (10.2)	196 (10.1)	201 (10.6)	193 (10.5)	190 (10.5)
Pho-Hak	504 (25.0)	489 (24.8)	477 (24.7)	471 (24.8)	447 (24.3)	452 (24.9)
**Age group (years), at enrollment (n; %)**						
1 - <5	200 (9.9)	190 (9.7)	187 (9.6)	183 (9.6)	183 (9.9)	178 (9.8)
5 - <10	398 (19.8)	397 (20.2)	389 (20.1)	384 (20.2)	379 (20.6)	374 (20.6)
10 - <15	500 (24.9)	495 (25.2)	490 (25.3)	478 (25.2)	461 (25.0)	460 (25.3)
15 - <25	400 (19.9)	385 (19.6)	369 (19.0)	361 (19.0)	343 (18.6)	335 (18.4)
25 - <35	200 (9.9)	190 (9.6)	191 (9.8)	185 (9.8)	178 (9.6)	170 (9.3)
35–55	314 (15.6)	311 (15.8)	307 (15.8)	306 (16.1)	298 (16.2)	297 (16.3)
History of self-reported fever in the serosurvey* (no. of subjects; %)	ND	322 (16.4)	428 (22.1)	398 (21.0)	342 (18.6)	281 (15.5)
Serosurvey subject also captured in the surveillance ** (no of subjects, % of acute fever episodes)	99	22 (6.8)	14 (3.3)	2 (0.5)	8 (2.3)	0
RT-PCR confirmed *** (n, % of the serosurvey subject also captured in the surveillance)	ND	3 (13.6)^ǀ^	6 (42.9)^ǁ^	0	4 (50.0)^ǂ^	0

* History of fever was based on self-report during the interval between visits (i.e. in the past ~6 months up to the point of subsequent visit)

** There were serosurvey subject who had fever and sought care for their febrile illness at Bang Phae Community Hospital (BPCH). These serosurvey subjects were also enrolled in the fever surveillance and their acute and convalescent samples were collected for investigation of dengue infection.

***There were serosurvey subjects who also participated in the passive fever surveillance implemented at BPCH in the same catchment area. Serosurvey subjects who sought care for febrile illness at BPCH during the interval between bleeds were enrolled in the surveillance and tested for acute dengue fever. The surveillance study data were presented separately [21]. These numbers represent those serosurvey subjects who had fever, participated in the surveillance, and were RT-PCR-confirmed with dengue in the surveillance.

ǀ1 secondary DENV-1 and 2 secondary DENV-2 cases were identified

ǁ1 primary DENV-1, 2 secondary DENV-1, and 3 secondary DENV-2 cases were identified

ǂ2 primary DENV-3, 1 secondary DENV-2, and 1 secondary DENV-3 cases were identified

### Indirect dengue ELISA test [24]

This antibody detection test was based on in-house indirect sandwich dengue IgG ELISA technique [24]. 96-well ELISA plates were filled with 100 μl/well of a diluted dengue monoclonal antibody 2H2 1:50 in 0.018 M carbonate buffer. After overnight incubation at 4°C, the plates were washed with 300 μl/well of 1% Tween 20 in phosphate buffer solution (PBS-T) and blocked with 5% skim milk in PBS-T for an hour at 37°C. After washing procedures, the plates were filled with 50 μl/well of an equal mixture of inhouse inactivated DENV-1-4 antigen. Fifty microliters per well of diluted serum/control with 1:400 with 5% skim milk in PBS-T were added in duplicate, and the plates were incubated at 37°C for an hour. After washing, the plates were filled with 50 μl/well of a goat-anti-human IgG antibody conjugated to horseradish peroxidase (KPL Inc., Gaithersburg, MD), diluted at 1:5000 in 5% skim milk PBS-T. After incubation at 37°C for an hour, the plates were washed and the substrate SureBlue TMP (KPL Inc., Gaithersburg, MD) was added. After incubation at room temperature for 30 minutes, the reaction was terminated by the addition of 50 μl of 0.2 M sulfuric acid and read at 450 nm using ELISA plate reader. For all enrolled subjects, we had IgG ELISA results for six visits and seropositivity was defined when the OD of the sample was higher by 2 times or greater compared to the OD of the negative control sample.

### 50% Dengue Plaque Reduction Neutralization Test (PRNT_50_)

On a subset (n = 640) of paired samples which showed a substantial rising of IgG titers over intervals, PRNT was performed to measure the neutralizing antibodies against DENV-1-4, as described previously [24,25]. PRNT_50_ titer was defined as the highest reciprocal serum dilution where the virus infectivity was reduced by 50% compared with control. In short, a monolayer of LLC-MK2 cells in M199 growth media was cultivated on the 12-well plates. The serum samples were inactivated (56°C for 30 minutes) and serially diluted to 1:10, 1:40. 1:160, 1:640 and 1:2560 by MEM diluent media. Each DENV serotype was then separately added into diluted serum and serum-virus mixtures were incubated at 35°C for an hour. The mixtures were then inoculated onto monolayer LLC-MK2 cells in 12-well plates. After four days of incubation at 35°C in 5% CO_2_, neutral red was used for straining the inoculated cells. Each sample was tested in duplicate, and the plaque-forming unit (pfu) was counted. PRNT_50_ titer was calculated by probit model with the SPSS program. When at least one of dengue serotypes titer was more than 15, it was considered PRNT_50_ seropositive. The threshold higher than 10 is used as a common standard, but we used 15 as a threshold to be more conservative and to consider for the background level of the test [26,27].

### Statistical analysis

A descriptive summary of characteristics is presented by visit number for all subjects (Table 1). Age was broken down to a 6-level categorical variable for descriptive purposes. Based on the distribution of the ratio of ELISA IgG titers (i.e. rising) between pairs, different values were explored to identify an accurate cut-off to identify potential DENV infection for selection of samples to undergo testing with PRNT_50_.

Among those samples with both PRNT_50 and_ IgG ELISA results, ELISA results were compared against PRNT_50_ as the gold standard and a IgG ELISA cut-off value was identified to demonstrate the best performance in terms of the sensitivity and specificity (Table 2). To report the population-level seroprevalence estimated in the study area, we compiled a descriptive summary of demographic and clinical characteristics for individuals determined to be seropositive (ELISA OD ≥ 2 times of negative control) for each bleed using the ELISA IgG titer cut-off (Table 3).

Also, among IgG negative individuals at enrollment, change in serostatus measured by indirect IgG ELISA was shown with proportions of sero-converted subjects over 6 bleeds. Main circulating serotypes were identified among these samples with no prior exposure to DENV. Based on PRNT results, the dominant serotype was defined as the one with the highest titer, greater than 1000, and when there are multiple serotypes with high titer values (greater than 100), it should be at least 2-fold difference. All analyses were performed using SPSS software version 18.0.

**Table 2:** Correlation between positivity of PRNT_50_ and dengue IgG using cut-off point at OD > 2.0 times of negative control.

		Dengue PRNT_50_		Total
		With prior exposure (defined by PRNT_50_ >15)	Dengue naïve (PRNT_50_ ≤ 15)	
**Sero status based on ELISA IgG**	**Classified as positive (IgG titer ≥2 negative control)**	748	16	764
	**Classified as negative (IgG titer <2 negative control)**	125	89	214
**Total**		873	105	978*

Sensitivity of test 0.86 (95% C.I: 0.83–0.88); Specificity of test 0.85 (95% C.I: 0.76–0.91); Positive predictive value 0.978 (95% C.I: 0.97–0.99); Negative predictive value 0.42 (95% C.I: 0.37–0.46); McNemar test P < 0.001

*We selected 640 paired samples which showed rising of dengue IgG titers in paired serum for further testing using in-house neutralization assay, PRNT_50_. However there were pairs that overlapped (i.e. there may be pairs that were selected and tested for visits 1–2 and then also for visits 2–3, then 3 samples were selected for PRNT testing). In the end, a total of 978 were tested with PRNT.

**Table 3:** Proportion of seropositivity (%) by residence (sub-district) and age at enrollment.

Seropositive* (n/N, %)	Visit 1	Visit 2	Visit 3	Visit 4	Visit 5	Visit 6
	M0 (Apr—May 2012)	M8 (Nov–Dec 2012)	M16 (Aug–Sep 2013)	M24 (Feb–Apr 2014)	M30 (Sep–Oct 2014)	M36 (Feb–Apr 2015)
**All subjects**	1494/2012 (74.3)	1513/1968 (76.8)	1496/1933 (77.4)	1455/1897 (76.7)	1426/1842 (77.4)	1440/1814 (79.4)
**Sub-district**						
Wang-Yen	261/348 (75.0)	263/341 (77.1)	259/334 (77.5)	243/319 (76.2)	236/318 (74.2)	242/307 (78.8)
Bang-Phae	226/301 (75.1)	227/294 (77.2)	228/291 (78.4)	228/292 (78.1)	235/284 (82.7)	233/275 (84.7)
Wat-Kaeo	174/250 (69.6)	186/247 (75.3)	180/243 (74.1)	169/231 (73.2)	165/228 (72.4)	166/222 (77.8)
Hau-Pho	179/254 (70.5)	193/248 (77.8)	191/242 (78.9)	180/236 (76.2)	178/227 (78.4)	176/225 (78.2)
Don-Kha	97/152 (63.8)	96/148 (64.9)	105/150 (70.0)	104/147 (70.7)	111/145 (76.6)	112/143 (78.3)
Don-Yai	134/203 (66.0)	134/201 (66.7)	128/196 (65.3)	129/201 (64.2)	126/193 (65.3)	121/190 (63.7)
Pho-Hak	423/504 (83.9)	414/489 (84.7)	405/477 (84.9)	402/471 (85.4)	375/447 (83.9)	390/452 (86.2)
**Enrollment age (years)**						
1 - <5	80/200 (40.0)	83/190 (43.7)	68/187 (36.3)	64/183 (35.0)	72/183 (39.3)	76/178 (42.7)
5 - <10	182/398 (45.7)	202/397 (50.9)	211/389 (54.2)	212/384 (55.2)	211/379 (55.6)	225/374 (60.1)
10 - <15	365/500 (73.0)	379/495 (76.6)	384/490 (78.4)	363/478 (75.9)	363/461 (78.7)	368/460 (80.0)
15 - <25	358/400 (89.5)	352/385 (91.4)	340/369 (92.1)	330/361 (91.4)	310/343 (90.4)	308/335 (91.9)
25 - <35	197/200 (98.5)	188/190 (98.9)	190/191 (99.4)	183/185 (98.9)	177/178 (99.4)	169/170 (99.4)
35–55	312/314 (99.4)	309/311 (99.4)	303/307 (98.7)	303/306 (99.0)	293/298 (98.3)	294/297 (99.0)

*Seropositive: ELISA OD ≥ 2 times of negative control

## Result

### Demographic data

During April and May 2012, 2012 residents of Bangphae district participated in the serosurvey enrollment bleed (M0). The ratio of male: female in our subjects was 0.8:1. After the rainy season (usually in July-October), the second visit (M8) was performed during November and December 2012, and 1968 subjects were followed up (Table 1).

Due to a minor study modification, subjects were invited to re-consent and 1933 subjects agreed to continue study participation in the third visit (M16), in August and September 2013. Between February and April 2014, 1897 subjects were followed up for the fourth visit (M24). For the fifth visit (M30; September to October 2014) and the last visit (visit 6; M36; February to April 2015), 1842 and 1814 subjects were followed up, respectively.

Of those subjects followed up in each serosurvey, between 15.5 and 22.1% of subjects with self-reported fever during the interval (Table 1). And of these with self-reported fever in the serosurvey, less than 7% sought care for their febrile illness at the facility under the surveillance study. These subjects who sought care also reported that they had a previous dengue infection over the interval, but this was not verified with information on medical charts. In the same catchment population in Bangphae district during an overlapping study period, there was a facility-based fever surveillance study [21]. These serosurvey subjects who sought care for febrile illness at BPCH during the interval between serosurvey visits were enrolled in our fever surveillance study and tested for acute dengue fever (with IgM/IgG ELISA and RT-PCR) [21]. There were few of these serosurvey subjects who were captured in the surveillance (Table 1). During the serosurvey interval between visits 1–2, 22 serosurvey subjects sought care for their febrile illness at BPCH and were enrolled in the surveillance, and 3 (1 secondary DENV-1 and 2 secondary DENV-2 cases) were dengue-confirmed with RT-PCR in the surveillance. Between visits 2–3, 14 serosurvey subjects were enrolled in the surveillance, and 6 (1 primary DENV-1, 2 secondary DENV-1, and 3 secondary DENV-2 cases) were dengue-confirmed with RT-PCR. Between visits 4–5, 8 serosurvey subjects were enrolled in the surveillance, and 4 (2 primary DENV-3, 1 secondary DENV-2, and 1 secondary DENV-3 cases) were dengue-confirmed with RT-PCR.

### Dengue seroprevalence

Based on the IgG ELISA results from all the samples, the OD ratios of ELISA IgG titers (i.e. rising) in paired samples were explored to identify an appropriate cut-off to identify potential DENV infection for selection of samples to undergo testing with PRNT_50_. This led to selection of 640 pairs of samples for PRNT_50_. With PRNT as the gold standard (50% dilution, titer >15), we used results of IgG ELISA and PRNT_50_ results on 640 pairs to explore different IgG ELISA titers (compared to negative control). We found that the ratio of 2.0 (between sample OD and negative control OD) to be the cut-off demonstrating the best combination of the high sensitivity, at 86% (95% C.I: 83–88%), and specificity, at 85% (95% C.I: 76%-91%) (Table 2).

Applying the cut-off ratio of 2.0 to all the samples, we calculated seroprevalence and reported seropositivity by age and region (Table 3). Based on indirect dengue ELISA, between 74.3 (at visit 1) and 79.4% (at visit 6) of subjects were found to be seropositive throughout the study period (Table 3). Residents in Pho-Hak showed higher proportions of DENV seropositivity, higher than 80%, compared to other sub-districts. Also, proportions of DENV seropositivity increased with age. Increasing trends of seropositivity were found over the study period in children aged between 5 and 10 years (45.7 to 60.1%) and among residents in Don-Kha (63.8 to 78.3%).

**Fig 1:**
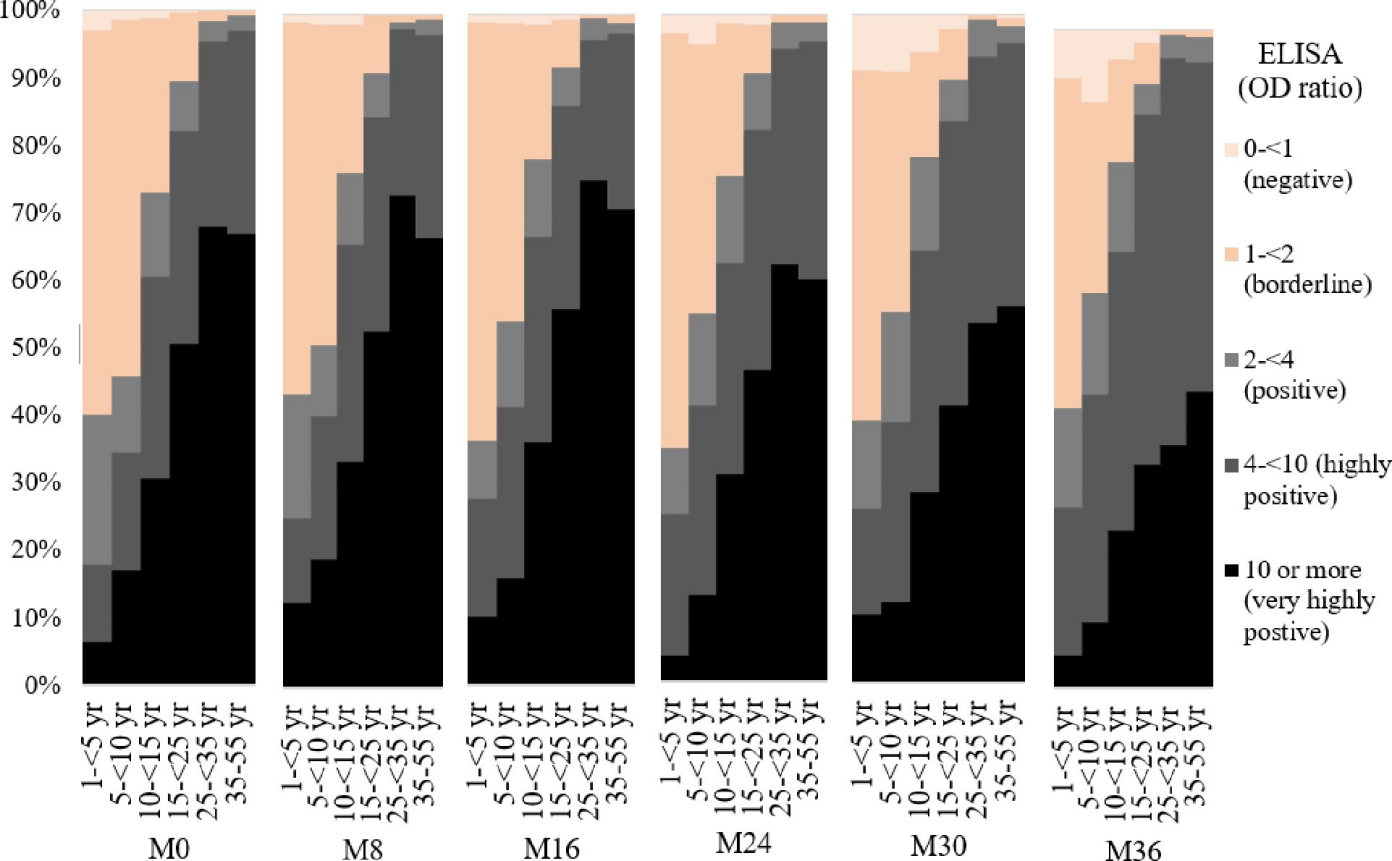
Proportion of dengue IgG seropositivity by age group for each serosurvey visit. **Legend:** Percentage of DENV seroprevalence based on indirect dengue IgG ELISA OD level. Axis X represent age group and the serosurvey visit Axis Y represent proportion of dengue IgG seropositivity.

Fig 1 showed proportion of seropositivity dengue IgG by age group at each serosurvey visit. It was shown that the rate of seropositivity increased sharply for subjects of school age and young adults. At M0 (visit 1), the odds ratio of subjects 10 years or older being seropositive (ELISA OD ratio) was 8.68 (95% CI 6.94–10.86, p <0.01), compared to those below 10 years. Furthermore, almost all of the subjects aged 25 years and older were seropositive and had higher ELISA titer than those younger than 25 years-of-age (Fig 1).

### The incidence of primary dengue infection

Using the same cut-off ratio of 2.0, among IgG negative individuals at baseline, there were 207 subjects who had seroconversion from IgG negative to positive over the entire study period. Given that we followed these IgG negative (ELISA OD < 2 times of negative control) subjects longitudinally over 6 serosurveys for changes in their IgG status, these cases are likely primary DENV infections. At enrollment, of 2012 subjects, IgG was positive in 1494 subjects (74.3%) (Tables 3 and 4). Of 518 subjects who were IgG negative at enrollment, there were 142 IgG seroconversions (27.4%) observed over the first 2 years of the study period between April 2012 (visit 1) and April 2014 (visit 4). At visit 4, there were 340 subjects found to be IgG negative. Of these, 65 subjects sero-converted by the end of study, over 1-year period, and annual incidence of primary dengue infection was estimated to be 19.1% between April 2014 (visit 4) and April 2015 (visit 6).

**Table 4:** Cascade of serostatus information detected by indirect ELISA among seronegative individuals at enrollment.

ELISA result											
Visit 1 (M0)		Visit 2 (M8)		Visit 3 (M16)		Visit4 (M24)		Visit 5 (M30)		Visit 6 (M36)	
(Apr-May 2012)		(Nov-Dec 2012)		(Aug-Sep 2013)		(Feb-Apr 2014)		(Sep-Oct 2014)		(Feb-Apr 2015)	
Negative (NEG)	518	NEG	431	NEG	373	NEG	340	NEG	286	NEG	251
										POS	23 (8.0%)
										ND	12
								POS	42 (12.4%)		
								ND	12		
						POS	18 (4.8%)				
						ND	15				
				POS	48 (11.1%)						
				ND	10						
		POS	76 (14.7%)								
		ND	11								
Positive (POS)	1494										
No data (ND)	0										

Negative (NEG): ELISA OD_450_ of sample /OD_450_ of Negative control < 2

Positive (POS): ELISA OD_450_ of sample /OD_450_ of Negative control ≥ 2

No data (ND) due to subjects’ missing visits

To identify the dominant circulating dengue serotype during this study period, all available pairs of PRNT_50_ whose baseline sample at enrollment was found to be negative on PRNT (n = 120) were evaluated. Overall, the proportion of DENV serotypes DENV-1:2:3:4 was found to be 6:30:15:9, respectively. During the study period, the dominant serotype was DENV-2 (40.5%), followed by DENV-3 (Table 5). In 2012, between V1-V2, DENV-3 was the most prevalent serotype in circulation, followed by DENV-2. In 2013 up to July, between V2-V3, DENV-2 was the most prevalent serotype in circulation. In 2014, between V3-V4 and V4-V5, DENV-2 was the most prevalent serotype in circulation, followed by DENV-4.

**Table 5:** DENV serotypes determined by PRNT_50_ in subjects with baseline PRNT_50_ negative*.

PRNT_50_	V1-V2	V2-V3	V3-V4	V4-V5	V5-V6
**No. of subjects with baseline PRNT**_**50**_ **negative**	**50**	**33**	**15**	**18**	**4**
DENV-1 dominant	3	2	0	1	0
DENV-2 dominant	10	12	4	4	0
DENV-3 dominant	12	2	0	0	1
DENV-4 dominant	3	0	4	2	0
Undetermined**	5	2	0	4	3
Not seroconverted (i.e. remaining seronegative)	17	15	7	7	0

Based on PRNT results, the dominant serotype was defined as the one with the highest titer greater than 1000 and when there are multiple serotypes with titers greater than 100, it should be at least 2-fold difference.

* PRNT_50_ negative (for all 4 serotype, titer ≤15) in the preceding sample in each pair

** undetermined: there is an evidence of rising titer between the pairs, but the dominant serotype was indistinguishable (i.e. more than 1 serotype without 2-fold difference)

First visit M0 (Apr-May 2012)

Second visit M8 (Nov-Dec 2012)

Third visit M16(Aug-Sep 2013)

Forth visit M24 (Feb-Apr 2014)

Fifth visit M30 (Sep- Oct 2014)

Sixth visit M36 (Feb-Apr 2015)

## Discussion

The burden of dengue is well documented in Thailand [7,8,16,17,28]. However, given the current context in terms of implementation of WHO recommendation for dengue vaccination, determining DENV serostatus is a key factor in consideration [29]. Such pre-screening tests should provide accurate results to minimize misclassification, and there are various measures of accuracy. Impact of sensitivity and specificity on positive predictive value may vary depending on the prevalence of the disease in a population [30,31]. Therefore, to report prevalence of previous infection by DENV measured by in-house IgG ELISA and document its diagnostic performance against a confirmatory method, PRNT, we conducted this cohort-based repeated serosurveys for longitudinal follow-up over a 3-year period.

This study revealed the level of seroprevalence in an area close to the sites of the Phase IIB and Phase III dengue vaccine trials in Ratchaburi, Thailand. Sampling for the subjects was stratified by age group and urbanicity of the neighborhood to obtain a catchment area population representative of the general population. The result showed high seroprevalence up to 77% on average for all ages and up to 98% among adults older than 25 years.

In terms of age, several studies documented changing epidemiologic patterns where a shift in age is observed with a higher association of dengue hemorrhagic fever with older ages [10,11]. Our data showed consistent results where seroprevalence was observed to increase sharply among subjects of school age and young adults.

Overall, our data support that there is high burden of dengue in this area. Although there are differences in terms of the time period, study area, laboratory confirmation methods, and age grouping, the seropositivity estimates (determined by PRNT_50_) in the Sanofi’s Phase IIB and Phase III dengue vaccine trials in Ratchaburi were similar to our findings, around 70%, especially among older children (9–17 years in the Sanofi’s trial data). However, among younger children (2–8 years in the Sanofi’s data), the seropositivity found in our study was lower, around 40%, compared to Sanofi’s data reported to be around 60% [32].

In our data with a 3-year follow up of the same subjects, we were able to assess primary DENV infection, defined by seroconversion from seronegative to seropositive based on indirect dengue IgG ELISA results in paired bleeds, among IgG negative subjects at baseline. Dengue hemorrhagic fever is usually related to the secondary heterotypic DENV infection, and the risk of severe dengue is known to be lower from the tertiary and quaternary infections [33]. Thus, incidence of primary DENV infection is relevant to determine the number of persons with a single prior exposure who might be at a risk of severe dengue. In this study, we demonstrated the incidence of primary DENV infection to be between 4.8–14.7% at different visit points during the study period and this could indicate the population at risk of severe outcomes of dengue upon subsequent infection. Also, our results on primary infections were also consistent with other previous reports. In the Sanofi’s Phase IIB and Phase III dengue vaccine trials, primary infection was reported in 24% of young children between 2–8 years-of-age and in 8.5% among older children between 9–17-years [32,33]. Although our study reports the proportion of primary infections across ages, our findings were similar with the primary infection occurrence reported in older children aged between 9–17 years in the Sanofi trials [32,33].

We were able to identify the serosurvey subjects who sought care for acute fever during the intervals between serosurveys in the fever surveillance study also conducted in the same catchment area. Of the serosurvey subjects, between 15% and 22% of subjects self-reported fever during the interval between visits (Table 1). And, of these, less than 7% had sought care for their febrile illness at BPCH, the facility under our surveillance (i.e. serosurvey subjects also captured in the fever surveillance). From these serosurvey subjects, acute and convalescent samples were obtained when they were enrolled in the surveillance as febrile patients, and the acute sample was tested with RT-PCR. There were only 3, 6, and 4 PCR-confirmed dengue cases in intervals between visits 1–2, 2–3, and 4–5, respectively. While we recognize the possibility of missing those serosurvey subjects who may have sought care elsewhere than the study facility, the study facility was the main healthcare provider covering the study area. Also, we did not verify these self-reported fever episodes with medical charts, at facilities other than the one under our surveillance study. This serosurvey was not designed to closely monitor clinical circumstances of the catchment area population. Therefore, we do not claim to have evaluated all possible dengue cases with fever from the longitudinal serosurvey cohort for their clinical outcomes. Furthermore, given that we identified seroconversion only among the 518 subjects without prior exposure to dengue, we do not attempt to associate self-reported dengue episodes with seroconversion.

To identify the dominant DENV serotype in circulation in the study area, we decided to focus on primary DENV infections with negative PRNT at baseline to avoid the issue of cross reactivity of antibodies among DENV serotypes after primary infection. For more than 40 years, all 4 DENV serotypes have been in circulation in Thailand, and the dominant serotype has changed unpredictably over time [7,34]. The finding showed that DENV-2 was most prevalent over the study period, and this finding was consistent to the information from the national survey where DENV-2 was the most prevalent serotype until 2015 [34]. In the BPCH-based fever surveillance project also conducted in an overlapping study period in Bang Phae district, most prevalent serotype in circulation was DENV-2 with some DENV-3 in 2012, DENV-3 in 2014, and DENV-4 & -3 in 2015. This was somewhat different from findings of our serosurveys where we found DENV-3 to be the most prevalent serotype in 2012, DENV-2 in 2013 and DENV-2 and -4 in 2014. In the surveillance study where subject recruitment was based on patients seeking care at the study facility, more than 70% of dengue-confirmed cases were between 5–19 years old. With more than 70% of subjects 10 years and older found to be seropositive in the serosurvey, the majority of those from whom we were able to identify serotypes with their baseline PRNT negative results (i.e. primary infection cases) would be children under 10 years of age. Furthermore, we had 46 serosurvey subjects enrolled in the concurrent fever surveillance, and few of them (n = 13, Table 1) were RT-PCR positive for DENV. Of these 13 subjects, 3 were dengue-confirmed with RT-PCR in 2012, between visits 1–2, and 1 was secondary DENV-1 and 2 were secondary DENV-2 cases. Additional 6 subjects were dengue-confirmed in 2013, between visits 2–3, and 1 was primary DENV-1, 2 and 3 were secondary DENV-1 and DENV-2 cases, respectively. In 2014, between visits 4–5, 4 subjects were dengue-confirmed in 2014 and 2 were primary DENV-3, with 1 secondary DENV-2 and 1 secondary DENV-3 cases. There was a small number of cases with serotyping information in the serosurvey. Nonetheless, in our data, the most common serotypes by PRNT did not match the most common serotype from the concurrent hospital-based surveillance. There is not much difference in terms of age of the dengue cases confirmed in the surveillance and in this serosurvey. A possible reason for the difference observed in serotype distribution in cases identified in the surveillance vs. the serosurvey could be due to the difference in sampled population (patients with dengue fever seeking for care vs. residents with mild or subclinical infection).

The main limitation of DENV serological evaluation is complexity associated with interpretation of laboratory results due to cross-reactivity of antibodies after primary infection. Antibodies to the recently exposed serotype may boost up the antibodies from the previously exposed dengue serotype or from other flaviviruses [35]. And, this may lead to misinterpretation of laboratory results, resulting in over-estimation of seroprevalence [36]. Japanese encephalitis virus (JEV) is endemic in Thailand, and there are reported outbreaks of Japanese encephalitis in different regions of the country [37]. Also, DENV cross-reactive antibodies could be induced by past JE vaccination, especially in the Expanded Program on Immunization (EPI) targeted population, among 12-month babies [38,39]. A limiting factor in this study was that it was not able to obtain validated information in terms of subjects’ history of JE vaccination. However, vaccine coverage of EPI in 2016 is reported to be high, around 90%. Therefore, there will be minimal misclassification due to cross-reactivity within the same age group.

The overall proportion of IgG positivity remained similar across the surveys, as shown in Table 3. However, given 6 repeated surveys following the same individuals, there were some participants lost to follow-up. The proportions of missed visit were 2% or less. However, there was a decrease in IgG positivity observed at visit 4. Without new recruitment, we reached out to subjects initially enrolled, even if they missed a visit, for subsequent visits. Therefore, if a sero-positive subject missed a visit on visit 4 but they returned for visit 5, this would lead to a decreased seropositive rates observed at the visit 4. In addition to the possibility of missed visits of sero-positive subjects, this may also be due to seroreversion (from seropositive to seronegative) or test errors.

In this study, with limited resources, we were only able to perform PRNT on a certain number (pairs) of samples. Therefore, we had applied a selection criteria to identify samples to undergo PRNT testing based on evidence of rising of IgG titers in pairs. We recognize that, if an individual was enrolled in the serosurvey with prior DENV infection but had not been re-infected during the study, then such samples will not be selected for PRNT given that their ratio of IgG titers will not demonstrate sufficient rising in pairs. Thus, our PRNT results may be biased toward seroconversion cases. However, we used PRNT results mostly to assess primary dengue cases and their serotypes.

Furthermore, we were able to longitudinally follow the changes in the DENV infection status, based on IgG, among seronegative subjects at baseline. Also, we recognize that seropositive individuals, especially primary infections, often have a lower IgG level and could have been misclassified as seronegative [40]. Therefore, some of false negative subjects in Table 2 could be cases of primary infections, and this could have led to underestimated seropositivity, measured by IgG ELISA. Such limitations have public health implications: the test may misclassify seropositive individuals with single prior exposure as being seronegative and would not receive the vaccine; and false positive individuals receiving the vaccine may have critical consequences (i.e. subsequent infection after vaccination could lead to severe disease). Furthermore, our study results may have limited generalizability to similar dengue hyper-endemic regions, such as Thailand.

Despite these limitations, this study employed a comprehensive laboratory algorithm where all samples were tested with dengue IgG ELISA and a selected number of samples were further tested with PRNT to obtain serostatus information over 6 repeated bleeds. PRNT is considered the gold standard and is a sensitive test for determining DENV infection. However, it was labor- and resource-intensive and time-consuming. Its interpretation requires technical expertise and tends to be complicated, especially after the secondary infection [14,24]. Performing ELISA is much simpler and less resource-intensive than performing PRNT; however, most of ELISA platforms are in-house developed and in absence of standardized information. In this study, we used the PRNT_50_ to guide how to interpret our ELISA result.

Our study findings provide updated evidence on the DENV serostatus in the studied population. In addition to documenting a high level seroprevalence in this study, we were also able to determine a proportion of primary infection cases in the studied population. Given that these cases with potentially single prior exposure may be at a risk of severe dengue, our data may give an estimate of the realistic demand of dengue vaccine, especially considering the importance of prior exposure to DENV and serostatus for the currently available dengue vaccine.

Furthermore, despite the fact that we used an in-house assay with limited validation [24], we demonstrated a high level of accuracy of IgG ELISA to determine DENV serostatus in serological evaluation. We recognize that the IgG ELISA used in this study was an in-house assay and is not available to be used in a non-research commercial setting for mass screening for pre-vaccination status. Nonetheless, the IgG ELISA assay used in this study showed reasonably high estimates of sensitivity at 86% and specificity of 85%. Given the fact that it is not a perfect test and there are still risks of vaccinating seronegative individuals, there should be further investigations to assess other serological assays to be used as a screening tool to determine DENV serostatus. In addition to this in-house assay, it would be ideal to test diagnostic accuracy of other readily available commercial kits for the purpose of screening prior to dengue vaccination. Furthermore, this study was not designed to monitor clinical circumstances of dengue. Additional longitudinal study with confirmatory tests and linking prevalence in the general population with complete evaluation of clinical dengue would help to further validate our findings. In addition, medical history of past infection (if known) should be included for more accurate evaluation of individual’s serostatus. Despite such shortcomings, our findings are relevant in terms of implications associated with use of dengue vaccines. Considering the current recommendations of WHO on the only licensed dengue vaccine, our findings provide evidence on a high level of diagnostic accuracy dengue IgG ELISA to determine dengue serostatus in policy development for dengue vaccine use, as well as for future studies on seroprevalence.

## Supporting information

S1 Strobe Checklist Dengue seroprevalence study in Bangphae district, Ratchaburi, Thailand: A cohort study in 2012–2015. (DOC)

S1 Table The data underlying the results presented in Fig 1 and Table 5. (DOCX)
