# Supplementary material for: Dengue virus seroprevalence study in Bangphae district, Ratchaburi, Thailand: A cohort study in 2012-2015
Source: PLoS Negl Trop Dis. 2022 Jan 4;16(1):e0010021. doi: 10.1371/journal.pntd.0010021 (PMC8726468; doi:10.1371/journal.pntd.0010021)
Supplement: S1 Table — (DOCX) [file pntd.0010021.s002.docx]

S1 Table

| ID | Visit | Date | D1 | D2 | D3 | D4 | Date | D1 | D2 | D3 | D4 | Interpretation |
| --- | --- | --- | --- | --- | --- | --- | --- | --- | --- | --- | --- | --- |
| 1 | V1-V2 | 29-Apr-12 | <10 | <10 | <10 | <10 | 24-Nov-12 | 1121 | 86 | 978 | 455 | DENV-1 dominant |
| 2 | V1-V2 | 29-Apr-12 | <10 | <10 | <10 | <10 | 25-Nov-12 | 643 | 308 | 220 | 133 | DENV-1 dominant |
| 3 | V1-V2 | 29-Apr-12 | <10 | <10 | <10 | <10 | 25-Nov-12 | 116 | <10 | <10 | <10 | DENV-1 dominant |
| 4 | V1-V2 | 29-Apr-12 | <10 | <10 | <10 | <10 | 23-Nov-12 | 41 | 463 | <10 | <10 | DENV-2 dominant |
| 5 | V1-V2 | 29-Apr-12 | <10 | <10 | <10 | <10 | 11-Dec-12 | 139 | 698 | <10 | 40 | DENV-2 dominant |
| 6 | V1-V2 | 30-Apr-12 | 14 | <10 | <10 | <10 | 22-Nov-12 | <10 | 127 | 60 | <10 | DENV-2 dominant |
| 7 | V1-V2 | 30-Apr-12 | <10 | <10 | <10 | <10 | 22-Nov-12 | 60 | 404 | 25 | 76 | DENV-2 dominant |
| 8 | V1-V2 | 30-Apr-12 | <10 | <10 | <10 | 14 | 21-Nov-12 | 225 | 871 | 57 | 220 | DENV-2 dominant |
| 9 | V1-V2 | 30-Apr-12 | <10 | <10 | <10 | 15 | 21-Nov-12 | 361 | 535 | 211 | 320 | DENV-2 dominant |
| 10 | V1-V2 | 30-Apr-12 | <10 | <10 | <10 | <10 | 21-Nov-12 | 247 | 492 | 379 | 377 | DENV-2 dominant |
| 11 | V1-V2 | 30-Apr-12 | <10 | <10 | <10 | <10 | 21-Nov-12 | 26 | 1012 | <10 | 18 | DENV-2 dominant |
| 12 | V1-V2 | 30-Apr-12 | <10 | <10 | <10 | <10 | 21-Nov-12 | 52 | 2389 | 89 | 310 | DENV-2 dominant |
| 13 | V1-V2 | 19-May-12 | 13 | <10 | <10 | 15 | 21-Nov-12 | 301 | >2560 | 17 | 346 | DENV-2 dominant |
| 14 | V1-V2 | 28-Apr-12 | <10 | <10 | <10 | <10 | 24-Nov-12 | 40 | 79 | 170 | 33 | DENV-3 dominant |
| 15 | V1-V2 | 29-Apr-12 | <10 | <10 | <10 | <10 | 23-Nov-12 | 97 | 11 | 295 | 132 | DENV-3 dominant |
| 16 | V1-V2 | 29-Apr-12 | <10 | <10 | <10 | <10 | 23-Nov-12 | 317 | 47 | 759 | 191 | DENV-3 dominant |
| 17 | V1-V2 | 29-Apr-12 | <10 | <10 | <10 | <10 | 23-Nov-12 | 322 | 25 | 601 | 151 | DENV-3 dominant |
| 18 | V1-V2 | 29-Apr-12 | <10 | <10 | <10 | <10 | 23-Nov-12 | 181 | 11 | 552 | 56 | DENV-3 dominant |
| 19 | V1-V2 | 29-Apr-12 | <10 | <10 | <10 | <10 | 25-Nov-12 | 361 | 190 | 1269 | 438 | DENV-3 dominant |
| 20 | V1-V2 | 30-Apr-12 | <10 | <10 | <10 | <10 | 22-Nov-12 | 60 | 15 | 668 | 275 | DENV-3 dominant |
| 21 | V1-V2 | 30-Apr-12 | <10 | <10 | <10 | <10 | 22-Nov-12 | 67 | <10 | 341 | 192 | DENV-3 dominant |
| 22 | V1-V2 | 30-Apr-12 | <10 | <10 | <10 | 11 | 22-Nov-12 | 196 | <10 | 706 | 199 | DENV-3 dominant |
| 23 | V1-V2 | 30-Apr-12 | <10 | <10 | <10 | <10 | 22-Nov-12 | 153 | 81 | 354 | 60 | DENV-3 dominant |
| 24 | V1-V2 | 30-Apr-12 | <10 | <10 | <10 | <10 | 22-Nov-12 | 131 | 305 | 432 | 230 | DENV-3 dominant |
| 25 | V1-V2 | 30-Apr-12 | <10 | 12 | <10 | <10 | 21-Nov-12 | 46 | 27 | 183 | 49 | DENV-3 dominant |
| 26 | V1-V2 | 28-Apr-12 | <10 | <10 | <10 | <10 | 24-Nov-12 | 132 | <10 | <10 | >2560 | DENV-4 dominant |
| 27 | V1-V2 | 30-Apr-12 | <10 | <10 | <10 | <10 | 22-Nov-12 | 504 | 339 | 331 | 895 | DENV-4 dominant |
| 28 | V1-V2 | 19-May-12 | 14 | <10 | <10 | <10 | 21-Nov-12 | 124 | 71 | 22 | 282 | DENV-4 dominant |
| 29 | V1-V2 | 29-Apr-12 | <10 | <10 | <10 | <10 | 23-Nov-12 | 1211 | 2514 | >2560 | 2100 | Undetermined |
| 30 | V1-V2 | 30-Apr-12 | 14 | <10 | <10 | <10 | 22-Nov-12 | 1016 | 1393 | 235 | 628 | Undetermined |
| 31 | V1-V2 | 30-Apr-12 | <10 | <10 | <10 | <10 | 22-Nov-12 | 1095 | 1493 | 1398 | 1353 | Undetermined |
| 32 | V1-V2 | 30-Apr-12 | <10 | <10 | <10 | <10 | 21-Nov-12 | 645 | 457 | 527 | 609 | Undetermined |
| 33 | V1-V2 | 30-Apr-12 | <10 | 11 | <10 | <10 | 21-Nov-12 | 2354 | >2560 | 2011 | 2513 | Undetermined |
| 34 | V1-V2 | 28-Apr-12 | 10 | <10 | <10 | <10 | 24-Nov-12 | 20 | <10 | 26 | <10 | No seroconverted |
| 35 | V1-V2 | 28-Apr-12 | <10 | <10 | <10 | <10 | 21-Nov-12 | 198 | 34 | 237 | 155 | No seroconverted |
| 36 | V1-V2 | 29-Apr-12 | <10 | <10 | <10 | <10 | 24-Nov-12 | <10 | <10 | 20 | 14 | No seroconverted |
| 37 | V1-V2 | 29-Apr-12 | <10 | <10 | <10 | <10 | 24-Nov-12 | 30 | <10 | 46 | 29 | No seroconverted |
| 38 | V1-V2 | 29-Apr-12 | <10 | <10 | <10 | <10 | 24-Nov-12 | <10 | <10 | <10 | <10 | No seroconverted |
| 39 | V1-V2 | 29-Apr-12 | <10 | <10 | <10 | <10 | 24-Nov-12 | 22 | <10 | 34 | <10 | No seroconverted |
| 40 | V1-V2 | 29-Apr-12 | 14 | <10 | <10 | <10 | 23-Nov-12 | 23 | <10 | 83 | 17 | No seroconverted |
| 41 | V1-V2 | 29-Apr-12 | <10 | <10 | <10 | <10 | 25-Nov-12 | <10 | <10 | <10 | <10 | No seroconverted |
| 42 | V1-V2 | 29-Apr-12 | <10 | <10 | <10 | <10 | 23-Nov-12 | <10 | <10 | 12 | <10 | No seroconverted |
| 43 | V1-V2 | 29-Apr-12 | <10 | <10 | <10 | <10 | 23-Nov-12 | 28 | <10 | 68 | <10 | No seroconverted |
| 44 | V1-V2 | 29-Apr-12 | <10 | <10 | <10 | <10 | 23-Nov-12 | 17 | <10 | 81 | <10 | No seroconverted |
| 45 | V1-V2 | 30-Apr-12 | <10 | <10 | <10 | <10 | 22-Nov-12 | <10 | 51 | <10 | 38 | No seroconverted |
| 46 | V1-V2 | 30-Apr-12 | <10 | <10 | <10 | <10 | 22-Nov-12 | 55 | <10 | <10 | 19 | No seroconverted |
| 47 | V1-V2 | 30-Apr-12 | 11 | <10 | <10 | <10 | 22-Nov-12 | 16 | 11 | 14 | 110 | No seroconverted |
| 48 | V1-V2 | 01-May-12 | 11 | 10 | <10 | <10 | 20-Nov-12 | <10 | <10 | <10 | 18 | No seroconverted |
| 49 | V1-V2 | 01-May-12 | <10 | <10 | <10 | <10 | 20-Nov-12 | <10 | 16 | <10 | <10 | No seroconverted |
| 50 | V1-V2 | 19-May-12 | <10 | <10 | <10 | <10 | 24-Nov-12 | <10 | <10 | <10 | <10 | No seroconverted |
| 51 | V2-V3 | 22-Nov-12 | <10 | <10 | <10 | <10 | 29-Aug-13 | 591 | 47 | 38 | 19 | DENV-1 dominant |
| 52 | V2-V3 | 22-Nov-12 | <10 | <10 | <10 | <10 | 29-Aug-13 | 306 | 82 | 63 | 62 | DENV-1 dominant |
| 53 | V2-V3 | 21-Nov-12 | <10 | <10 | <10 | <10 | 31-Aug-13 | 16 | 350 | 20 | 29 | DENV-2 dominant |
| 54 | V2-V3 | 21-Nov-12 | <10 | <10 | <10 | <10 | 30-Aug-13 | 328 | 1400 | 262 | 496 | DENV-2 dominant |
| 55 | V2-V3 | 21-Nov-12 | <10 | <10 | <10 | <10 | 30-Aug-13 | 420 | 1738 | 341 | 495 | DENV-2 dominant |
| 56 | V2-V3 | 21-Nov-12 | 11 | <10 | <10 | <10 | 01-Sep-13 | 25 | 836 | 41 | 68 | DENV-2 dominant |
| 57 | V2-V3 | 21-Nov-12 | <10 | <10 | <10 | <10 | 30-Aug-13 | 20 | 328 | 82 | 54 | DENV-2 dominant |
| 58 | V2-V3 | 21-Nov-12 | 12 | <10 | <10 | <10 | 30-Aug-13 | 37 | 485 | 63 | 189 | DENV-2 dominant |
| 59 | V2-V3 | 21-Nov-12 | <10 | <10 | <10 | <10 | 30-Aug-13 | 145 | 787 | 153 | 221 | DENV-2 dominant |
| 60 | V2-V3 | 21-Nov-12 | <10 | <10 | <10 | <10 | 30-Aug-13 | 24 | 860 | 28 | 18 | DENV-2 dominant |
| 61 | V2-V3 | 24-Nov-12 | <10 | <10 | <10 | <10 | 01-Sep-13 | 106 | 567 | 49 | 46 | DENV-2 dominant |
| 62 | V2-V3 | 24-Nov-12 | <10 | <10 | <10 | <10 | 01-Sep-13 | 231 | 893 | 80 | 167 | DENV-2 dominant |
| 63 | V2-V3 | 24-Nov-12 | <10 | <10 | <10 | <10 | 01-Sep-13 | 86 | 523 | 39 | 167 | DENV-2 dominant |
| 64 | V2-V3 | 20-Nov-12 | 13 | <10 | <10 | <10 | 30-Aug-13 | 182 | 436 | 107 | 295 | DENV-2 dominant |
| 65 | V2-V3 | 23-Nov-12 | <10 | <10 | <10 | <10 | 11-Sep-13 | 184 | 122 | 304 | 142 | DENV-3 dominant |
| 66 | V2-V3 | 22-Nov-12 | <10 | <10 | <10 | <10 | 29-Aug-13 | 77 | 124 | 274 | 59 | DENV-3 dominant |
| 67 | V2-V3 | 25-Nov-12 | <10 | <10 | <10 | <10 | 01-Sep-13 | >2560 | >2560 | 1095 | 1563 | Undetermined |
| 68 | V2-V3 | 21-Nov-12 | <10 | <10 | <10 | <10 | 28-Aug-13 | 99 | 131 | 142 | 19 | Undetermined |
| 69 | V2-V3 | 24-Nov-12 | <10 | <10 | <10 | <10 | 01-Sep-13 | 86 | <10 | 16 | 19 | No seroconverted |
| 70 | V2-V3 | 23-Nov-12 | <10 | <10 | <10 | <10 | 27-Aug-13 | <10 | <10 | <10 | <10 | No seroconverted |
| 71 | V2-V3 | 23-Nov-12 | 13 | <10 | <10 | <10 | 27-Aug-13 | 123 | 26 | 140 | 25 | No seroconverted |
| 72 | V2-V3 | 22-Nov-12 | <10 | 11 | <10 | <10 | 29-Aug-13 | 36 | 181 | 29 | 40 | No seroconverted |
| 73 | V2-V3 | 21-Nov-12 | <10 | <10 | <10 | 12 | 18-Sep-13 | <10 | <10 | <10 | <10 | No seroconverted |
| 74 | V2-V3 | 21-Nov-12 | <10 | <10 | <10 | <10 | 28-Aug-13 | <10 | <10 | <10 | <10 | No seroconverted |
| 75 | V2-V3 | 21-Nov-12 | <10 | <10 | <10 | <10 | 28-Aug-13 | <10 | 40 | <10 | <10 | No seroconverted |
| 76 | V2-V3 | 21-Nov-12 | <10 | <10 | <10 | <10 | 28-Aug-13 | <10 | <10 | <10 | <10 | No seroconverted |
| 77 | V2-V3 | 20-Nov-12 | <10 | <10 | <10 | <10 | 31-Aug-13 | 28 | 77 | 28 | 24 | No seroconverted |
| 78 | V2-V3 | 20-Nov-12 | <10 | <10 | <10 | <10 | 31-Aug-13 | 27 | <10 | <10 | <10 | No seroconverted |
| 79 | V2-V3 | 23-Nov-12 | <10 | 10 | <10 | <10 | 27-Aug-13 | 63 | 92 | <10 | 22 | No seroconverted |
| 80 | V2-V3 | 21-Nov-12 | <10 | <10 | <10 | <10 | 28-Aug-13 | 42 | 62 | 52 | 77 | No seroconverted |
| 81 | V2-V3 | 22-Nov-12 | <10 | <10 | <10 | <10 | 29-Aug-13 | <10 | <10 | <10 | <10 | No seroconverted |
| 82 | V2-V3 | 24-Nov-12 | <10 | <10 | <10 | <10 | 01-Sep-13 | <10 | <10 | <10 | <10 | No seroconverted |
| 83 | V2-V3 | 21-Nov-12 | <10 | <10 | <10 | <10 | 30-Aug-13 | <10 | 73 | <10 | <10 | No seroconverted |
| 84 | V3-V4 | 29-Aug-13 | <10 | <10 | <10 | <10 | 20-Feb-14 | 645 | 911 | 526 | 683 | DENV-2 dominant |
| 85 | V3-V4 | 30-Aug-13 | <10 | <10 | <10 | 11 | 21-Feb-14 | 192 | 583 | 369 | 158 | DENV-2 dominant |
| 86 | V3-V4 | 31-Aug-13 | <10 | <10 | <10 | <10 | 23-Feb-14 | 229 | 603 | 165 | 354 | DENV-2 dominant |
| 87 | V3-V4 | 31-Aug-13 | <10 | 13 | <10 | <10 | 22-Feb-14 | 802 | 1892 | 805 | 436 | DENV-2 dominant |
| 88 | V3-V4 | 27-Aug-13 | <10 | <10 | <10 | <10 | 19-Feb-14 | 100 | 185 | 62 | 713 | DENV-4 dominant |
| 89 | V3-V4 | 27-Aug-13 | <10 | <10 | <10 | <10 | 19-Feb-14 | 27 | 126 | 19 | 269 | DENV-4 dominant |
| 90 | V3-V4 | 29-Sep-13 | <10 | <10 | <10 | <10 | 23-Feb-14 | 531 | 507 | 352 | 988 | DENV-4 dominant |
| 91 | V3-V4 | 31-Aug-13 | <10 | <10 | <10 | <10 | 22-Feb-14 | 960 | 802 | 484 | 1031 | DENV-4 dominant |
| 92 | V3-V4 | 30-Aug-13 | <10 | <10 | <10 | <10 | 21-Feb-14 | <10 | 19 | 95 | <10 | No seroconverted |
| 93 | V3-V4 | 10-Sep-13 | 14 | <10 | <10 | <10 | 27-Feb-14 | 59 | 51 | <10 | <10 | No seroconverted |
| 94 | V3-V4 | 01-Sep-13 | <10 | <10 | <10 | 12 | 23-Feb-14 | 10 | 10 | <10 | 22 | No seroconverted |
| 95 | V3-V4 | 27-Aug-13 | 11 | 12 | <10 | <10 | 19-Feb-14 | 17 | 62 | <10 | 11 | No seroconverted |
| 96 | V3-V4 | 30-Aug-13 | <10 | <10 | <10 | <10 | 22-Feb-14 | <10 | <10 | <10 | <10 | No seroconverted |
| 97 | V3-V4 | 31-Aug-13 | <10 | <10 | <10 | <10 | 22-Feb-14 | <10 | 40 | <10 | <10 | No seroconverted |
| 98 | V3-V4 | 01-Sep-13 | <10 | <10 | <10 | <10 | 23-Feb-14 | <10 | <10 | <10 | <10 | No seroconverted |
| 99 | V4-V5 | 19-Feb-14 | <10 | <10 | <10 | 11 | 12-Sep-14 | 1730 | 526 | 293 | 996 | DENV-1 dominant |
| 100 | V4-V5 | 23-Feb-14 | <10 | <10 | <10 | <10 | 14-Sep-14 | 311 | 808 | 586 | 450 | DENV-2 dominant |
| 101 | V4-V5 | 20-Feb-14 | <10 | <10 | <10 | 11 | 12-Sep-14 | 120 | 451 | 91 | 100 | DENV-2 dominant |
| 102 | V4-V5 | 22-Feb-14 | <10 | <10 | <10 | <10 | 13-Sep-14 | 2067 | >2560 | 888 | 1961 | DENV-2 dominant |
| 103 | V4-V5 | 22-Feb-14 | <10 | <10 | <10 | <10 | 13-Sep-14 | 1709 | 2041 | 1480 | 1266 | DENV-2 dominant |
| 104 | V4-V5 | 23-Feb-14 | <10 | <10 | <10 | <10 | 14-Sep-14 | 672 | 1211 | 2395 | >2560 | DENV-4 dominant |
| 105 | V4-V5 | 19-Feb-14 | <10 | <10 | <10 | <10 | 12-Sep-14 | 844 | 814 | 484 | 1085 | DENV-4 dominant |
| 106 | V4-V5 | 23-Feb-14 | <10 | 15 | <10 | <10 | 14-Sep-14 | >2560 | >2560 | >2560 | 870 | Undetermined |
| 107 | V4-V5 | 21-Feb-14 | 10 | 14 | <10 | <10 | 10-Sep-14 | 155 | 173 | 26 | 56 | Undetermined |
| 108 | V4-V5 | 21-Feb-14 | <10 | 13 | <10 | <10 | 10-Sep-14 | 72 | 215 | 127 | 156 | Undetermined |
| 109 | V4-V5 | 20-Feb-14 | <10 | <10 | <10 | <10 | 11-Sep-14 | 159 | 315 | 226 | 78 | Undetermined |
| 110 | V4-V5 | 21-Feb-14 | <10 | 13 | <10 | <10 | 10-Sep-14 | 11 | 32 | 42 | 13 | No seroconverted |
| 111 | V4-V5 | 23-Feb-14 | <10 | <10 | <10 | <10 | 14-Sep-14 | <10 | 25 | <10 | <10 | No seroconverted |
| 112 | V4-V5 | 23-Feb-14 | <10 | <10 | <10 | <10 | 14-Sep-14 | <10 | 114 | <10 | 38 | No seroconverted |
| 113 | V4-V5 | 22-Feb-14 | <10 | 10 | <10 | <10 | 14-Sep-14 | 19 | 24 | 0 | <10 | No seroconverted |
| 114 | V4-V5 | 22-Feb-14 | <10 | <10 | <10 | <10 | 13-Sep-14 | <10 | <10 | <10 | <10 | No seroconverted |
| 115 | V4-V5 | 22-Feb-14 | <10 | <10 | <10 | <10 | 13-Sep-14 | 32 | 91 | 14 | <10 | No seroconverted |
| 116 | V4-V5 | 22-Feb-14 | <10 | <10 | <10 | <10 | 13-Sep-14 | 11 | <10 | 93 | <10 | No seroconverted |
| 117 | V5-V6 | 13-Sep-14 | <10 | 10 | <10 | <10 | 14-Feb-15 | 219 | 589 | 920 | 198 | DENV-3 dominant |
| 118 | V5-V6 | 14-Sep-14 | <10 | 10 | <10 | <10 | 15-Feb-15 | 40 | 235 | 131 | 28 | Undetermined |
| 119 | V5-V6 | 13-Sep-14 | <10 | <10 | <10 | <10 | 14-Feb-15 | 65 | 163 | 128 | 122 | Undetermined |
| 120 | V5-V6 | 14-Sep-14 | <10 | <10 | <10 | <10 | 15-Feb-15 | 22 | 189 | 164 | 277 | Undetermined |
